# Impacto da COVID-19 na Vida do Cardiologista e Cirurgião Cardiovascular Brasileiros

**DOI:** 10.36660/abc.20201231

**Published:** 2021-11-01

**Authors:** Andre Luiz Cerqueira Almeida, Marcelo Melo, Rodrigo Elton Ferreira Rodrigues, Luis Fábio Botelho, Paulo André Abreu Almeida, Silvio Henrique Barberato

**Affiliations:** 1 Santa Casa de Misericórdia de Feira de Santana - Cardiologia Feira de Santana BA Brasil Santa Casa de Misericórdia de Feira de Santana - Cardiologia, Feira de Santana, BA - Brasil; 2 Sociedade Brasileira de Cardiologia Departamento de Imagem Cardiovascular São Paulo SP Brasil Sociedade Brasileira de Cardiologia Departamento de Imagem Cardiovascular, São Paulo, SP - Brasil; 3 Universidade Federal da Paraíba João Pessoa PB Brasil Universidade Federal da Paraíba, João Pessoa, PB - Brasil; 4 UNIFACS Curso de Medicina Salvador BA Brasil UNIFACS Curso de Medicina, Salvador, BA - Brasil; 5 Sociedade Brasileira de Cardiologia Diretoria de Qualidade Assistencial Rio de Janeiro RJ Brasil Sociedade Brasileira de Cardiologia - Diretoria de Qualidade Assistencial, Rio de Janeiro, RJ - Brasil; 6 CardioEco - Centro de Diagnóstico Cardiovascular Curitiba PR Brasil CardioEco - Centro de Diagnóstico Cardiovascular, Curitiba, PR - Brasil

**Keywords:** COVID-19, Coronavirus-19, Pandemia, Cardiologistas, Cirurgiões, Doenças Cardiovasculares, Fatores de Risco, Sistemas de Saúde, Infecção/complicações, Profissionais de Saúde, Comportamento Sedentário, Epidemiologia

## Introdução

A pandemia da COVID-19 (sigla do inglês *Coronavirus Disease* - 2019) impactou significativamente os serviços de cardiologia. O número de consultas, exames e intervenções cardiológicas diminuiu em várias partes do mundo nos últimos meses.1,2 Contudo, apesar da pressão crescente e da carga sobre o sistema de saúde, a oferta de serviços em cardiologia não foi interrompida, já que doença cardiovascular preexistente coloca os pacientes sob maior risco de infecção, complicações e a manifestações cardíacas primárias da COVID-19.^3^

Além disso, os efeitos da COVID-19 têm afetado a sociedade em geral e os profissionais de saúde em particular, a saber: impacto na saúde física e mental, perturbações financeiras e alterações na qualidade de vida.^3-5^ Sendo assim, a pandemia causou verdadeira ruptura em diversos aspectos da prática profissional e da vida de médicos e demais profissionais de saúde.^1,2,5^

Nosso estudo visou avaliar o impacto causado pela pandemia de COVID-19 na vida dos médicos(as) cardiologistas e cirurgiões(ãs) cardiovasculares brasileiros(as), considerando questões ligadas à atividade profissional, renda, saúde e estilo de vida.

## Material e Métodos

Os autores disponibilizaram e divulgaram um formulário online no site da Sociedade Brasileira de Cardiologia (SBC) e no site da Diretoria de Qualidade Assistencial da SBC, convidando os médicos especialistas em cardiologia a participarem. Adicionalmente foram enviados convites por meio de aplicativo de mensagem amplamente disponível para grupos de cardiologistas de sociedades regionais, departamentos e grupos de estudo pertencentes à SBC. Esta participação foi voluntária e secreta, não havendo a opção do cardiologista se identificar. Não houve qualquer compensação financeira ou material como retorno à participação na pesquisa. O período da coleta de dados foi de 10 de julho de 2020 a 22 de julho de 2020. O formulário online (https://wdcom.typeform.com/report/fmQda3LQ/tOStzUhXlifR8JPj) consistiu em 28 perguntas com preenchimento obrigatório, sobre a prática assistencial e a qualidade de vida do cardiologista brasileiro durante a pandemia da COVID-19. A maioria das questões foi do tipo múltipla escolha, sendo que em muitas delas era possível responder mais de uma opção.

### Aspectos éticos

Seguindo a recomendação da Resolução 510 do Conselho Nacional de Saúde, este questionário não foi encaminhado para avaliação pelo sistema CEP/CONEP, visto tratar-se de uma pesquisa de opinião pública com participantes não identificados.

### Análise estatística

Foi realizada a análise descritiva dos dados obtidos na amostra. As variáveis nominais ou categóricas foram descritas por seus valores absolutos, percentagens ou proporções. As variáveis numéricas foram descritas como média e desvio-padrão ou mediana e intervalo interquartil, a depender do padrão de distribuição. O teste exato de Fisher foi utilizado para testar associações entre variáveis categóricas, utilizando um nível de significância de 5%. A análise dos dados, assim como a construção dos gráficos, foi feita com o auxílio do Excel®, Microsoft 365®. As análises inferenciais foram feitas utilizando o programa estatístico *Stata/SE* versão 16.1, desenvolvido pela StataCorp®.

## Resultados

### Aspectos gerais

**Figura 1:**
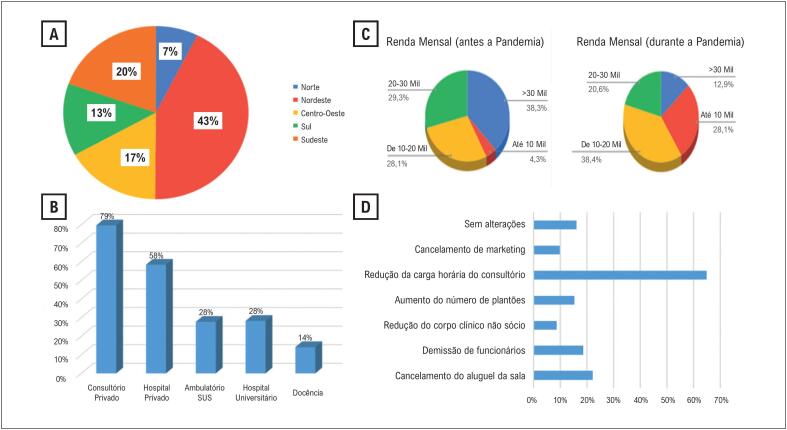
Distribuição dos cardiologistas participantes do estudo por região geográfica (A), local de trabalho (B); renda mensal antes e durante a pandemia (C), e reestruturação no trabalho devido à pandemia (D).

Um total de 1224 cardiologistas acessaram o questionário. Destes, dois recusaram a participação e 1222 responderam, representando 9,4% dos cardiologistas adimplentes na SBC. A média de idade da população do estudo foi 47,9 ± 11,5 anos; 711 (58,2%) do sexo masculino. A Figura 1 mostra a distribuição dos respondentes por região do Brasil (1A), seus locais de trabalho (1B), a renda mensal antes e durante a pandemia (1C) e a reestruturação imposta à rotina de trabalho dos cardiologistas (1D).

Observou-se uma associação significativa (p<0,001) entre sexo masculino e maiores faixas de renda (Tabela 1). Cardiologistas que trabalham no setor privado ou têm atividade de docência tiveram maior mudança de renda durante a pandemia (p<0,001).

**Tabela 1:** Associação entre as variáveis avaliadas na pesquisa sobre o impacto da pandemia da COVID-19 na vida de cardiologistas brasileiros (n=1222)

Variável 01	Variável 02	Associação (valor de p)
Sexo feminino	Redução de atividade sexual	< 0,001
Sexo masculino	Maiores faixas de renda	< 0,001
Trabalho no setor privado	Maior mudança de renda	< 0,001
Atividade de docência	Maior mudança de renda	< 0,001
Idade < 50 anos	Aumento de plantões	< 0,001
Subespecialidade ecocardiografia	Redução na atividade física	< 0,001
Renda mensal	Redução na atividade física	> 0,05
Ganho de peso	Redução na atividade física	> 0,05
Sexo	Redução na atividade física	> 0,05
Sexo	Mudanças na rotina do trabalho	> 0,05
Faixa etária	Medidas adotadas para reduzir os custos	> 0,05

### Aspectos relacionados à renda e ao trabalho

Houve um aumento de 37,5% no número de cardiologistas que passaram a trabalhar em três ou mais plantões por semana durante a pandemia. Por outro lado, 64% reduziram a carga horária no consultório, 22% cancelaram aluguel de sala de consultório, 18% precisaram demitir funcionários e 9% cancelaram investimentos em marketing (Figura 1-D).

**Figura 2:**
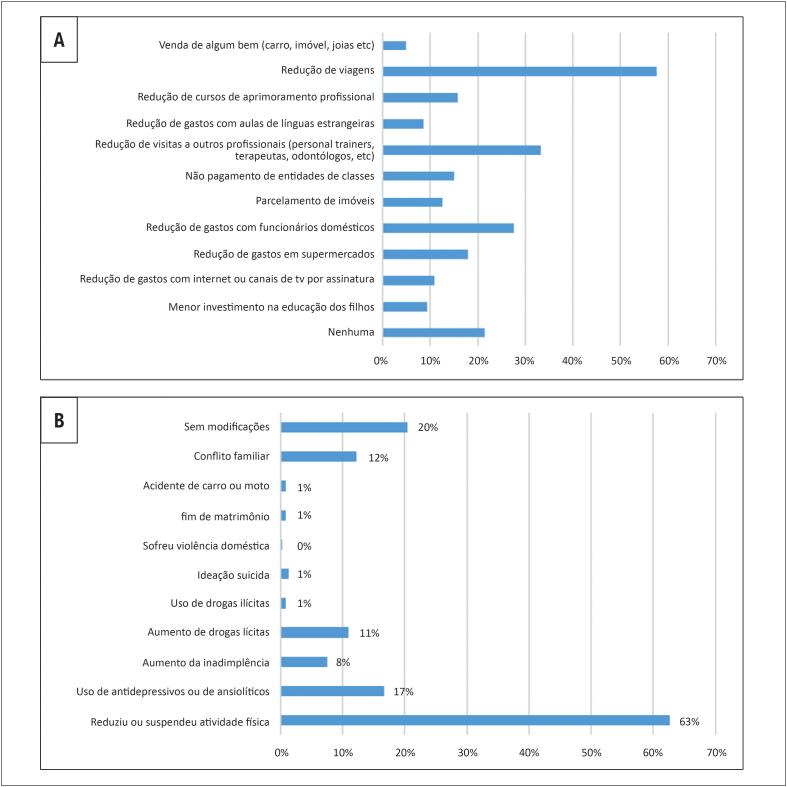
Medidas de redução de custos (A) mudanças no estilo de vida (B) durante a pandemia da COVID-19 relatadas por cardiologistas brasileiros.

Como reflexo da redução do retorno financeiro durante a pandemia, 15% dos cardiologistas deixaram de pagar entidades de classe. Outras medidas para redução de custos estão expressas na Figura 2A.

Quando analisamos o impacto da pandemia por faixa etária, considerando 50 anos como ponto de corte, dos resultados válidos, 56% dos entrevistados tinham menos de 50 anos e 44% tinham 50 anos ou mais. Desses dois grupos, observamos um aumento (p<0,001) no número de plantões entre os médicos mais jovens, sem impactar na renda média entre os dois grupos.

**Figura 3:**
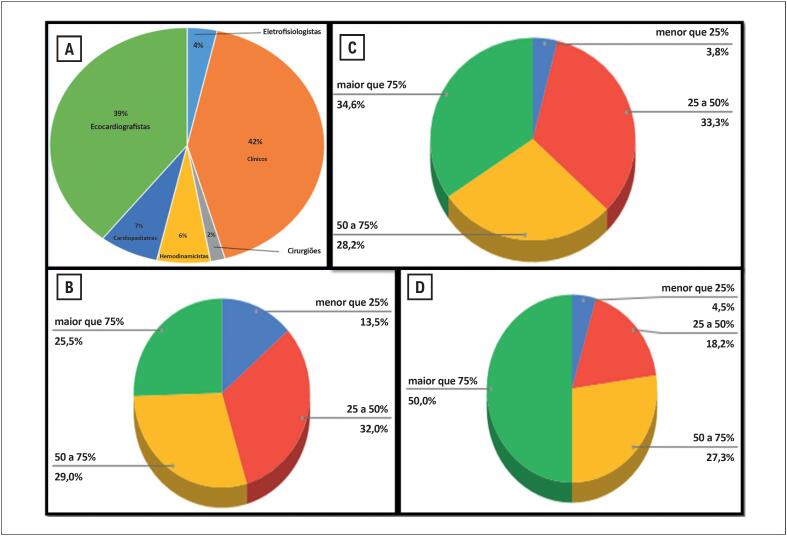
Distribuição dos cardiologistas participantes (n=1222) por subespecialidade (A); e por porcentagem de profissionais que relataram redução nos procedimentos de ecocardiografia (B); hemodinâmica (C) e cirurgia cardíaca (D)

Cardiologistas clínicos representaram 42% da amostra, seguidos por ecocardiografistas (39%), cardiopediatras (7%), hemodinamicistas (6%), eletrofisiologistas (4%) e cirurgiões cardiovasculares (2%) (Figura 3A). Dentre os ecocardiografistas, 54,5% constataram uma redução superior a 50% no volume de exames realizados/mês durante a pandemia (Figura 3B). Na hemodinâmica, 62,8% dos entrevistados relataram redução maior que 50% no volume de exames ou procedimentos no mesmo período (Figura 3-C). Entre os cirurgiões cardiovasculares, 77,3% relataram redução superior a 50% no número de cirurgias (Figura 3-D). A subespecialidade ecocardiografia mostrou associação com redução na prática de atividade física pelos profissionais (p <0,001).

**Figura 4:**
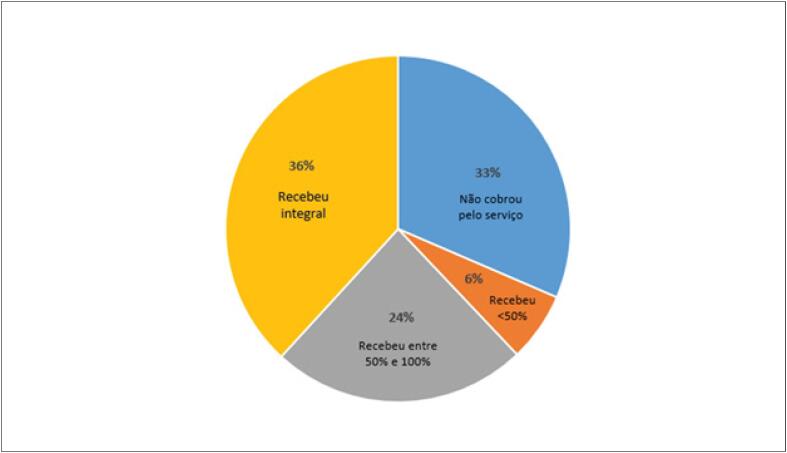
Reembolso das consultas por videoconferência realizadas durante a pandemia da COVID-19 por cardiologistas brasileiros (n=1222).

**Figura 5:**
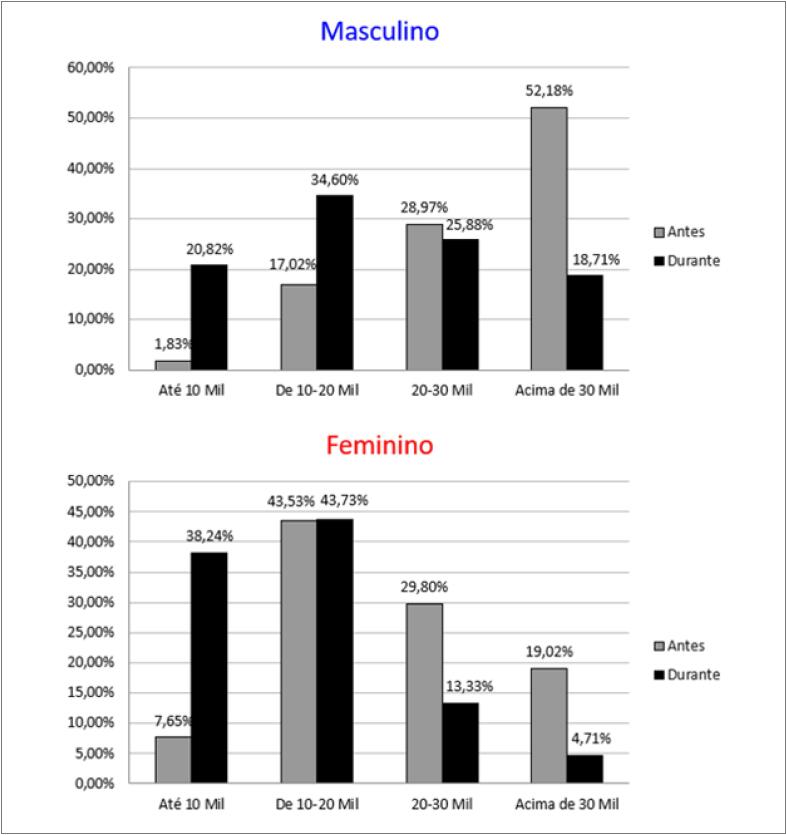
Distribuição dos cardiologistas brasileiros (n=1222) quanto à renda e sexo antes e durante a pandemia da COVID-19.

As consultas por videoconferência no âmbito da Telemedicina foram autorizadas recentemente no Brasil. Nesta pesquisa, 30% dos entrevistados as realizaram, porém apenas 36% foram reembolsados integralmente pelo serviço (Figura 4). Antes da pandemia, 48,8% das mulheres ganhavam mais de R$20 mil ao mês e, durante a pandemia, houve uma redução de 63%, e apenas 18% continuaram com essa renda. Entre os homens a redução foi de 45% (81,2% para 44,6%) (Figura 5). Apenas 7,6% das mulheres e 1,8% dos homens ganhavam menos de R$10mil por mês antes da pandemia, e esse número passou para 38,2% e 20,8%, respectivamente, durante a pandemia (Figura 5).

**Figura 6:**
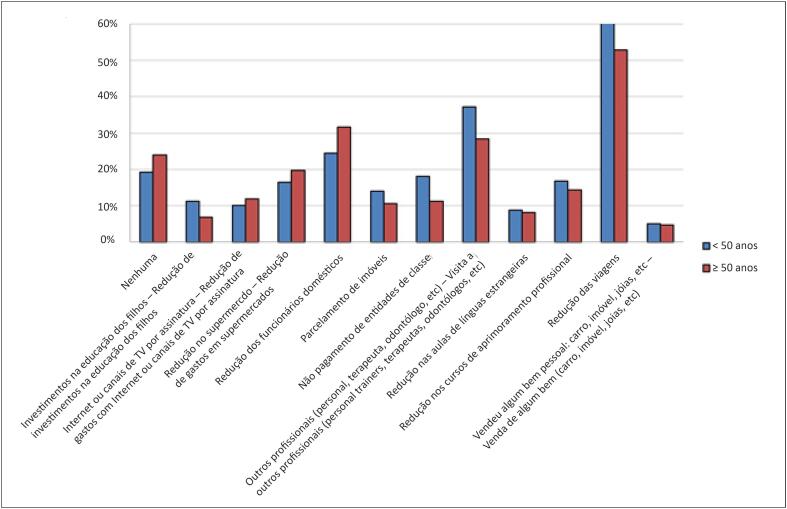
Relação da faixa etária com as medidas para redução de custos durante a pandemia.

As medidas adotadas para reduzir os custos durante a pandemia não tiveram associação significativa com a faixa etária da amostra (Figura 6).

### Aspectos relacionados às mudanças de rotina e de estilo de vida

Dos entrevistados, 69% praticavam atividade física antes da pandemia. Desses, 63% reduziram ou suspenderam a prática da atividade física durante a pandemia. Doze por cento experimentaram conflito familiar (quatro relatos de violência doméstica); 17% passaram a fazer uso de antidepressivos ou ansiolíticos e 11% aumentaram o uso de drogas lícitas (Figura 2B). Não houve associação de redução da prática de atividade física com o sexo ou com a renda (p >0,05).

Considerando as últimas quatro semanas da pandemia, 44% dos entrevistados relataram ganho de peso, sendo que 13% relataram ganho superior a 3 kg. Em 35% dos casos, o peso manteve-se estável. Neste mesmo período de observação, 26% relataram aumento do consumo de bebidas alcoólicas, enquanto 30% referiram que o consumo permaneceu estável. Não houve associação entre ganho de peso e mudança na atividade física (p >0,05).

**Figura 7:**
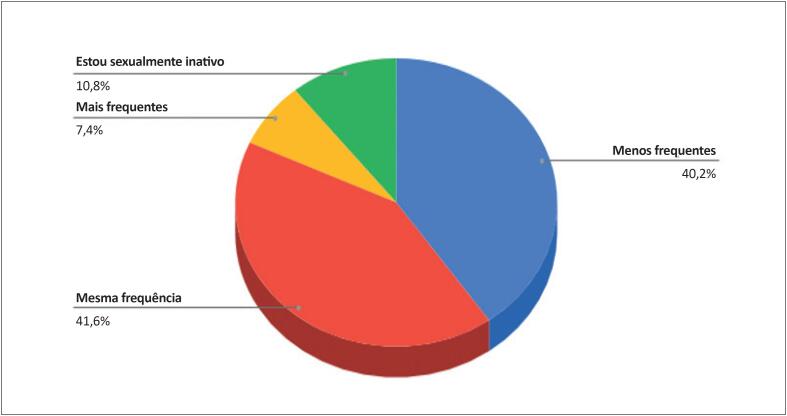
Frequência de relações sexuais relatadas pelos cardiologistas durante a pandemia (n=1222)

Dos entrevistados, 40,2% relataram diminuição na frequência das relações sexuais, para 41,6% essa frequência manteve-se estável, e apenas 7,4% relataram aumento na frequência de relações (Figura 7). Essa redução foi mais significativa nos profissionais do sexo feminino (p <0,001).

### Aspectos relacionados à infecção pela COVID-19

Do total de investigados, 54,9% mostraram moderada ou muita preocupação em trabalhar na linha de frente do combate à COVID-19. Até o final do período pesquisado (22/07/2020), 20% dos cardiologistas entrevistados tinham tido infecção sintomática confirmada pelo novo coronavírus. Em 1,8% dos casos, os sintomas foram graves, necessitando internamento, ao passo que os sintomas foram leves e sem necessidade de hospitalização em 15% dos que responderam ao questionário. Em 3% dos casos a infecção foi confirmada, mas a evolução foi assintomática.

## Discussão

O presente estudo relata os resultados da primeira pesquisa nacional que avaliou o impacto causado pela pandemia da COVID-19 nas questões profissionais, financeiras, de saúde (física e mental), e de estilo de vida dos médicos cardiologistas brasileiros. As respostas de 1222 cardiologistas, distribuídos por todas as regiões do Brasil, demonstraram um forte impacto em todas as áreas investigadas. Foi nítida a redução nos ganhos financeiros, associada à redução da carga horária no consultório e à necessidade de aumento no número de plantões semanais. Como consequência, a quitação de alguns compromissos financeiros ficou comprometida, incluindo pagamento de entidade de classe, cursos de aprimoramento profissional e custeio da educação dos filhos. Notamos ainda uma importante redução na prática de atividades físicas e de relações sexuais durante a pandemia, além de aumento nos conflitos familiares e no uso de antidepressivos e ansiolíticos. Quase metade dos cardiologistas relataram aumento no peso corporal e 25% relataram aumento na ingestão de bebidas alcoólicas.

Assim como aconteceu com os cardiologistas brasileiros, uma pesquisa divulgada pela British Medical Association em julho de 2020 apontou que 39,5% dos médicos britânicos relataram redução nos ganhos financeiros e 30,7% referiram condições de saúde mental relacionadas ou agravadas pelo seu trabalho durante a pandemia da COVID-19, como depressão, ansiedade, estresse, esgotamento e sofrimento emocional.^4^ Em recente pesquisa realizada com 766 urologistas brasileiros, 54,8% relataram redução nos ganhos financeiros superior a 50% durante a pandemia da COVID-19, 32,9% relataram ganho de peso, 60,0% redução na prática de atividade física, 39,9% aumentaram o consumo de álcool e 34,9% referiram redução na atividade sexual.^5^

Vários níveis de evidências sugerem que a inatividade física pode provocar importantes repercussões na fisiologia cardiovascular.^6^ As atividades físicas dos cardiologistas estudados foram reduzidas ou suspensas em 63% dos casos, o que pode ter impactado nos 44% que tiveram aumento de peso superior a 3kg. Uma publicação recente relacionou a redução de atividade física e ganho de peso com o aumento do risco de doenças cardiovasculares, além de alertar sobre outros perigos da obesidade.^7^

Pelo nosso levantamento, 26% passaram a ingerir mais bebidas alcoólicas, enquanto 40% dos entrevistados afirmaram uma diminuição no número de relações sexuais em comparação a antes da pandemia. É plausível a associação do aumento do consumo de álcool e da exacerbação de conflitos familiares com o impacto psicológico causado pelo isolamento social prolongado. O aumento de conflitos familiares foi relatado por 12% dos nossos entrevistados, incluindo quatro profissionais que sofreram violência doméstica. Esse número pode ser bem maior, posto que houve aumento desses casos durante o período de isolamento social em outros países como a China (onde os casos triplicaram),^8^ Reino Unido, Estados Unidos e França (atingindo 36%).^9^ No Brasil, esse índice chegou a aumentar em 17%, conforme dados do Ministério da Mulher.^10^

O governo brasileiro regularizou e autorizou temporariamente o atendimento remoto de pacientes por meio da telemedicina no Brasil.^11^ Mesmo em fase ainda inicial, precipitada pelo isolamento social imposto pela pandemia, 30% dos cardiologistas que responderam ao questionário afirmaram ter realizado teleconsultas, embora apenas 36% destes tenham sido reembolsados integralmente pelo serviço. Como comparação, 38,7% dos urologistas brasileiros relataram ter realizado teleconsultas, com mais de 50% informando reembolso pelo serviço prestado.^5^

Em 2017, um questionário foi enviado via e-mail a todos os 13 462 cardiologistas adimplentes associados à SBC; 2101 (15,6%) responderam efetivamente, sendo 1509 (71,8%) homens e 592 (28,2%) mulheres.^12^ Dos 1222 (9,1% dos sócios da SBC) que responderam ao nosso questionário, 711 (58,2%) eram homens. A faixa etária foi semelhante aos respondedores das duas enquetes, sendo que 51,3% dos que responderam à pesquisa da SBC tinham mais de 50 anos, contra 44% dos respondedores do nosso trabalho. Em relação à distribuição geográfica, 54% dos sócios adimplentes da SBC estão na região Sudeste, 19% no Nordeste, 15% no Sul, 8% na região Centro-Oeste e 3% no Norte. Dentre os que responderam ao nosso questionário, 43% estavam na região Nordeste, 17% no Centro-Oeste, 13% no Sul, 20% no Sudeste e 7% no Norte do Brasil.

O presente estudo possui algumas limitações inerentes aos estudos transversais baseados em resposta a um questionário. O número de respondentes da atual pesquisa representa pouco menos de 10% do número de cardiologistas associados à SBC. A distribuição geográfica dos participantes da pesquisa é diferente da dos sócios da SBC. Outro ponto relevante é a impossibilidade de comprovar as respostas ou esclarecê-las; porém, apesar da incerteza da veracidade das respostas, o estudo foi coerente com outros dados publicados em âmbito nacional e internacional. Embora nosso estudo tenha encontrado algumas associações interessantes e com significância estatística, tais achados devem ser considerados meramente exploratórios, não podendo desconsiderar a possibilidade de achados falso-positivos pela quantidade de testes de hipóteses realizados.

## Conclusão

Esse estudo demonstra o impacto negativo da pandemia de COVID-19 no trabalho, renda, saúde e estilo de vida dos médicos cardiologistas brasileiros. São dados de extrema relevância que ajudarão no planejamento em futuros cenários de caos como o atual enfrentamento pandêmico.
