# Supplementary material for: Impacto da COVID-19 na Vida do Cardiologista e Cirurgião Cardiovascular Brasileiros
Source: Arq Bras Cardiol. 2021 Nov 1;117(5):1048–55. [Article in Portuguese] doi: 10.36660/abc.20201231 (PMC8682098; doi:10.36660/abc.20201231)
Supplement: Translation

# Translation: Impact of COVID-19 on the Life of Brazilian Cardiologists and Cardiovascular Surgeons

**Authors:** Andre Luiz Cerqueira Almeida, Marcelo Melo, Rodrigo Elton Ferreira Rodrigues, Luis Fábio Botelho, Paulo André Abreu Almeida, Silvio Henrique Barberato

**Affiliations:** 1 Feira de Santana BA Brazil Santa Casa de Misericórdia de Feira de Santana - Cardiologia, Feira de Santana, BA - Brazil; 2 São Paulo SP Brazil Sociedade Brasileira de Cardiologia Departamento de Imagem Cardiovascular, São Paulo, SP - Brazil; 3 João Pessoa PB Brazil Universidade Federal da Paraíba, João Pessoa, PB - Brazil; 4 Salvador BA Brazil UNIFACS Curso de Medicina, Salvador, BA - Brazil; 5 Rio de Janeiro RJ Brazil Sociedade Brasileira de Cardiologia - Diretoria de Qualidade Assistencial, Rio de Janeiro, RJ - Brazil; 6 Curitiba PR Brazil CardioEco - Centro de Diagnóstico Cardiovascular, Curitiba, PR - Brazil

**Keywords:** COVID-19, Coronavirus-19, Pandemics, Cardiologists, Surgeons, Cardiovascular Diseases, Risk Factors, Health Systems, Infection/complications, Health Personnel, Sedentary Behavior, Epidemiology

## Introduction

The COVID-19 (from the English coronavirus disease) pandemic has had a strong impact on cardiology services. The number of visits and cardiac interventions has decreased in many parts of the world in recent months.^1,2^ However, despite increasing pressure and burden on the healthcare system, the provision of care has continued, particularly because patients with pre-existing cardiovascular disease are at high risk of COVID-19 infection, complications, and primary cardiac manifestations.^3^

In addition, effects of the COVID-19 have affected the society and in particularly healthcare professionals in terms of physical and mental health consequences, financial disturbances, and changes in quality of life.^3-5^ Therefore, the COVID-19 pandemic has affected many aspects of the work and life of health care providers.^1,2,5^

Our study aimed at assessing the impact of the COVID-19 pandemic on the life of cardiologists and cardiac surgeons in Brazil, regarding professional practice, income, health and life style.

## Methods

The authors released an online form on two websites, the Brazilian Society of Cardiology (SBC) website and on the SBC quality of care board website, to invite cardiologists to participate in the research. In addition, an invitation was sent by a freeware, cross-platform centralized instant messaging service to groups of cardiologists and local societies, departments and study groups, members of the SBC. Participation was voluntary and necessarily anonymous. Participants did not receive any financial or material compensation. Data were collected from 10 July to 22 July 2020. The online form was composed of 28 questions of mandatory completion about healthcare practices and the quality of life of Brazilian cardiologists during the COVID-19 pandemic. Most questions were multiple-choice questions, and many of them had more than one possible answers.

### Ethical aspects

A descriptive analysis of data was performed - nominal or categorical variables were expressed as absolute values, percentages or proportions. Numerical variables were expressed as mean and standard deviation or median and interquartile range, according to the data distribution. The Fisher's exact test was used to assess possible associations between categorical variables, at a level of significance of 5%. The Microsoft 365 Excel software was used for data analysis and construction of graphs. The inferential analysis of data was performed using Stata/SE 16.1 (StataCorp).

## Results

### General aspects

A total of 1,224 cardiologists accessed the questionnaire; two declined to participate and 1,222 answered the instrument, corresponding to 9.4% of current members of the SBC. Mean age of the study population was 47.9 ± 11.5 years, 711 (58.2%) were men. Figure 1 shows the distribution of respondents by Brazilian geographical region (1-A), workplace (1B), monthly income before and during the pandemic (1C) and changes in work routines of the cardiologists (1D).

**Figure 1:** Distribution of Brazilian cardiologists participating in the study (n=1,222) by geographical area (A) place of work (B), monthly income before and during the COVID-19 pandemic (in Brazilian reals) (C), and changes in work routines / structure due to the pandemic.

There was a significant association between male sex and higher income ranges (p<0.001) (Table 1). Cardiologists who work in the private sector or in teaching activities experienced greater changes in income during the pandemic (p<0.001).

**Table 1:** Table 1 Associations between the variables analyzed in the assessment of the COVID-19 pandemic on the life of Brazilian cardiologists (n=1,222)

Variable 01	Variable 02	Association (p-value)
Female sex	Reduction in sexual activity	< 0.001
Male sex	Higher income ranges	< 0.001
Work in the private sector	Greater changes in income	< 0.001
Teachinig activities	Greater changes in income	< 0.001
Age < 50 years	Working more shifts	< 0.001
Work as an echocardiography sonographer	Decrease in physical activity	< 0.001
Monthly income	Decrease in physical activity	> 0.05
Weight gain	Decrease in physical activity	> 0.05
Sex	Decrease in physical activity	> 0.05
Sex	Changes in work routines	> 0.05
Age range	Measures to reduce costs	> 0.05

### Aspects of work and income

The number of cardiologists who started to work at three or more shifts per week during the pandemic increased by 37.5%. On the other hand, 64% reduced their work hours at the office, 22% canceled their office lease, 18% had to dismiss employees, and 9% canceled investments in marketing (Figure 1D).

As a consequence of the income reduction, 15% of cardiologists stopped paying medical councils of which they were members. Other measures for cost reduction are described in Figure 2A.

**Figure 2:** Measures for cost reduction (A) and changes in lifestyle (B) during the COVID-19 pandemic reported by Brazilian cardiologists (n=1,222).

When we analyzed the impact of the pandemic by age range, considering 50 years of age as the cutoff, considering the valid responses, 56% of respondents were younger than 50 years and 44% aged 50 years or more. In these two groups, we found a significant increase (p<0.001) in the number of work shifts per week among the younger group, with no significant impact on the monthly income in none of the groups.

Forty-two percent of our sample were clinical cardiologists, 39% echocardiographists, 2% cardiovascular surgeons (Figure 3A). Among the echocardiographists, 54.5% reported a reduction greater than 50% in the volume of tests performed per month during the pandemic (Figure 3B). In the field of hemodynamics, 62.8% of respondents reported a reduction greater than 50% in the volume of tests or procedures in the same period (Figure 3C). Among cardiovascular surgeons,77.3% reported a reduction greater than 50% in the number of surgeries (Figure 3D). A significant association was found between working in the field of echocardiography and a reduction in physical activity by professionals (p<0.001).

**Figure 3:** Distribution of cardiologists participating in the study by subspecialties (A) and percentages of professionals that reported a reduction in echocardiographic procedures (B); hemodynamic procedures (C) and cardiac surgeries (D).

Telemedicine videoconferencing has been recently approved in Brazil. This modality of consultation was used by 30% of respondents in our study; however, 36% of these were fully reimbursed for the service (Figure 4). Before the pandemic, 48.8% of the women gained more than R$20,000 thousand a month and, during the pandemic, there was a reduction of 63%, and only 18% maintained the same income. Among men, 81.2% and 44.6% gained more than R$20,000 thousand a month before and during the pandemic, i.e., a reduction of 45% (Figure 5). Only 7.6% of women and 1.8% of men gained less than R$10,000 a month before the pandemic, and this percentage increased to 38.2% and 20.8%, respectively, during the pandemic (Figure 5).

**Figure 4:** Reimbursement of consultations by video technology made during the COVID-19 pandemic, reported by Brazilian cardiologists (n=1,222).

**Figure 5:** Distribution of Brazilian cardiologists (n=1,222) by monthly income (in Brazilian reals) and sex before and during the COVID-19 pandemic.

No association was found between measures for cost reductions adopted during the pandemic and age ranges (Figure 6).

**Figure 6:** Relationship between age range and measures of cost reduction during the pandemic (n=1,222).

### Changes in work routines and lifestyle

Among the respondents, 69% reported being physically active before the pandemic, and 63% of them reduced or stopped physical activities during the pandemic. Twelve percent of participants experienced family conflicts (four reported domestic violence); 17% started using antidepressants or anxiolytics, and 11% increased the use of illicit drugs (Figure 2B). No association was found between reduction in physical activity with sex or income (p>0.05).

Considering the last four weeks of the pandemic, 44% of respondents reported weight gain, and 13% reported gaining more than three kilograms; weight was stable in 35% of respondents. In the same period of analysis, 26% of respondents reported increased alcohol consumption and 30% reported a stable consumption. There was no association between weight gain and change in physical activity (p>0.05).

In addition, for 40.2% and 41.6% of respondents, the frequency of sexual intercourse was reduced or unchanged, respectively, during the pandemic, and only 7.4% reported an increase in this frequency (Figure 7). The reduction in the frequency of sexual activity was more pronounced in women than men (p<0.001).

**Figure 7:** Frequency of sexual activity during the pandemic reported by Brazilian cardiologists (n=1,222).

### Aspects of the COVID-19 infection

In our sample, 54.9% of cardiologists reported to be moderately or very concerned about being on the frontline against COVID-19. Until the end of the study period (07/22/2020), 20% of respondents had a symptomatic COVID-19 infection; 1.8% of them had severe symptoms and required hospitalization, and 15% of respondents had mild symptoms and did not require hospitalization. Three percent of the cases had a confirmed infection, with an asymptomatic course though.

## Discussion

The present study describes the results of the first nationwide survey that assessed the impact of the COVID-19 pandemic on professional and financial lives, health (physical and mental), and lifestyle of Brazilian cardiologists. The responses from 1,222 cardiologists from all geographical regions of the country demonstrated a strong impact of the pandemic on all these aspects. The findings reviewed a marked decrease in financial gain, associated with a reduction in working hours at the office, and an increase in the number of shifts per week. Consequently, payment of some expenses was affected, including payment of medical councils, attendance in professional development courses, and payment of school fees of their children. In addition, there was an important reduction in the practice of physical activities, frequency of sexual intercourse, and an increase in family conflicts and use of antidepressant and anxiolytics. Almost half of cardiologists reported weight gain and 25% reported an increase in alcohol consumption.

Similar to the results obtained here, a study published by the British Medical Association in July 2020 showed that 39.5% of British doctors reported a reduction in financial earnings, and 30.7% reported mental health conditions relating to or made worse by work during the COVID-19 pandemic, such as depression, anxiety, stress, burnout, and emotional distress.^4^ In a recent study conducted with 766 Brazilian urologists, 54.8% reported a reduction greater than 50% in the income during the pandemic, 32.9% reported weight gain, 60.0% reported a reduction in physical activity, 39.9% increased alcohol consumption, and 34.9% reported a reduction in sexual activity.^5^

Several lines of evidence have suggested that physical inactivity may have important repercussions in cardiovascular physiology.^6^ Physical activity was reduced or stopped by 63% of respondents, which may have contributed to the weight gain greater than three kilograms reported by 44% of participants. A recent publication established a relationship of physical activity reduction and weight gain with increased risk of cardiovascular diseases and drew attention to other obesity-related health problems.^7^

Our data showed that 26% of respondents increased alcohol consumption, and 40% reported a reduction in sexual activity during the pandemic as compared with before the pandemic. It is plausible to associate the increased alcohol consumption and exacerbation of family conflicts with the psychological impact caused by prolonged social isolation. An increase in family conflicts was reported by 12% of respondents, including four professionals who experienced domestic violence. It is of note that this number may be much higher, considering that the number of domestic violence incidents has drastically increased in other countries during the period of social isolation, including China (where the number of cases has tripled),^8^ the United Kingdom, the United States and France (reaching a 36% increase).^9^ In Brazil, the incidence increased by 17% according to the Brazilian Ministry of Women.^10^

The Brazilian government has regulated and temporarily authorized the remote consultation of patients by telemedicine in Brazil.^11^ Precipitated by the social isolation imposed by the COVID-19 pandemic, and still in its early stages, 30% of the cardiologists reported using teleconsultation, although only 36% of them were fully reimbursed for the service. This differs from the results of another study that showed that 38.7% of the Brazilian urologists participating in the study reported evaluating their patients by teleconsultation, and 50% of them were fully reimbursed for the service.^5^

In 2017, a questionnaire was sent by e-mail to all 13,462 cardiologists, active members of the SBC; 2,101 (15.6%) responded to the questionnaire, 1509 (71.8%) men and 592 (28.2%) women.^12^ In our study, of 1,222 (9.1% of the SBC active members) of respondents, 711 (58.2%) were men. Age range of respondents was similar between the two surveys, but 51.3% of respondents to the first survey were older than 50 years, versus 44% in the current study. Regarding the geographical distribution, 54% of active members of the SBC live in the southeast region of Brazil, 19% live in the northeast, 15% in the south, 8% in the middle-west, and 3% in the north region of Brazil. Among respondents, 43% live in the northeast region, 17% in the middle-west, 13% in the south. 20% in the southeast and 7% in the north.

The present study has some limitations inherent to cross-sectional studies based on questionnaire responses. The number of respondents represent slightly less than 10% of the number of cardiologists members of the SBC. The geographical distribution of respondents is markedly different from that of the members of the SBC. Also, it was impossible to prove or elucidate the questionnaire responses; however, although the truthfulness of the responses could not be checked, the study was consistent with literature published in Brazil and in other countries. In addition, although we have found some interesting and even statistically significant associations, our results should be considered exploratory, and the possibility of false-positive results cannot be disregarded due to the number of hypothesis tests performed.

## Conclusion

This study reinforces the negative impact of the COVID-19 pandemic on the work, income, health, and lifestyle of Brazilian cardiologists. We present extremely relevant data that will help in planning in future scenarios of chaos, like the current challenge of the COVID-19 pandemic.
